# Assessment of De-Escalation of Empirical Antimicrobial Therapy in Medical Wards with Recognized Prevalence of Multi-Drug-Resistant Pathogens: A Multicenter Prospective Cohort Study in Non-ICU Patients with Microbiologically Documented Infection

**DOI:** 10.3390/antibiotics13090812

**Published:** 2024-08-27

**Authors:** Vasiliki Rapti, Garyfallia Poulakou, Anastasia Mousouli, Athanasios Kakasis, Stamata Pagoni, Evmorfia Pechlivanidou, Aikaterini Masgala, Styliani Sympardi, Vasileios Apostolopoulos, Charalampos Giannopoulos, Nikolaos Alexiou, Kostoula Arvaniti, Christina Trakatelli, Apostolos Prionas, Michael Samarkos, George L. Daikos, Helen Giamarellou

**Affiliations:** 1 3rd Department of Internal Medicine and Laboratory, Sotiria General Hospital, School of Medicine, National and Kapodistrian University of Athens, 15772 Athens, Greece; gpoulakou@gmail.com; 2 3rd Department of Internal Medicine, “G. Gennimatas” General Hospital of Athens, 11527 Athens, Greecekakasisathan@gmail.com (A.K.);; 3 Department of Hygiene, Epidemiology and Medical Statistics, Medical School, National & Kapodistrian University of Athens, 15772 Athens, Greece; 4 2nd Department of Internal Medicine, Konstantopouleio General Hospital, 14233 Athens, Greece; 5 1st Department of Internal Medicine, Thriasio General Hospital of Eleusis, 19600 Attica, Greece; 6 Intensive Care Unit, Papageorgiou University Affiliated Hospital, 56403 Thessaloniki, Greece; arvanitik@hotmail.com; 7 3rd Department of Internal Medicine, Medical School, Aristotle University of Thessaloniki, 54124 Thessaloniki, Greece; ctrak@auth.gr; 8 1st General Surgery Department, Papageorgiou General Hospital, Aristotle University of Thessaloniki, 54124 Thessaloniki, Greece; 9 1st Department of Internal Medicine, Medical School, National and Kapodistrian University of Athens, 15772 Athens, Greece; 10 1st Department of Internal Medicine-Infectious Diseases, Hygeia General Hospital, 15772 Athens, Greece; e.giamarellou@hygeia.gr

**Keywords:** antimicrobial stewardship programs, antimicrobial de-escalation, MDR pathogens, medical wards, feasibility, safety, efficacy, mortality

## Abstract

Antimicrobial resistance poses a major threat to human health worldwide and the implementation of antimicrobial stewardship programs (ASPs), including antimicrobial de-escalation (ADE), is a multifaceted tool for minimizing unnecessary or inappropriate antibiotic exposure. This was a prospective observational study of 142 non-Intensive Care Unit (ICU) patients with microbiologically documented infection who were initially administered empirical antimicrobial therapy and admitted to the medical wards of 6 tertiary-care hospitals in Greece from January 2017 to December 2018. Patients were divided into two groups, the ADE and non-ADE group, based on whether ADE was applied or not, respectively. Exploratory end-points were ADE feasibility, safety and efficacy. ADE was applied in 76 patients at a median time of 4 days (IQR: 3, 5). An increased likelihood of ADE was observed in patients with urinary tract (OR: 10.04, 95% CI: 2.91, 34.57; *p* < 0.001), skin and soft tissue (OR: 16.28, 95% CI: 1.68, 158.08; *p* = 0.016) and bloodstream infections (OR: 2.52, 95% CI: 1, 6.36; *p* = 0.05). Factors significantly associated with higher rates of ADE were clarithromycin administration, diagnosis of urinary tract infection (UTI), isolation of *E. coli*, age and symptoms type on admission. Mortality was lower in the ADE group (18.4% vs. 30.3% *p* < 0.1) and ADE was not significantly associated with the probability of death (*p* = 0.432). ADE was associated with favorable clinical outcomes and can be performed even in settings with high prevalence of multi-drug resistant (MDR) pathogens without compromising safety.

## 1. Introduction

The World Health Organization (WHO) has declared antimicrobial resistance (AMR) as one of the top ten public health threats affecting humanity worldwide [1]. The global burden associated with AMR was an estimated 4.95 million deaths in 2019, of which 1.27 million were directly attributable to drug resistance [2]. In 2015, recognizing the urgent need to combat AMR, the World Health Assembly endorsed a global action plan, with one of the five strategic objectives outlined being the optimization of the use of antimicrobial medicines in human and animal health, thereby recommending the implementation of ASP programs [3]. Antimicrobial stewardship (AMS), which encompasses the coordinated interventions designed to improve and measure the appropriate use of antimicrobial agents in terms of selection, dose, route, and duration of administration, is now recognized as an essential tool for minimizing inappropriate or unnecessary antibiotic use and reducing both AMR rates and associated healthcare costs [4,5,6]. ADE is a key component of ASP and refers to the discontinuation of one or more components of combination empirical therapy and/or the change from a broad-spectrum to a narrower spectrum antimicrobial [7,8]. ADE feasibility and efficacy are strongly correlated with various factors, such the relevance of diagnosis, early and adequate collection of microbiological samples, pathogen isolation, appropriateness of empirical treatment, lower baseline severity, or clinical resolution at the time of culture positivity [8,9].

Early administration of empirical antimicrobial therapy in patients with suspected infection is crucial and results in favorable outcomes [10]. Notably, the impact is even greater in patients with confirmed or suspected sepsis, and the Surviving Sepsis Campaign recommends the early administration of empirical broad-spectrum antimicrobial regimens, ideally within the first hour of patient clinical assessment [11,12,13]. However, this practice can lead to antimicrobial overconsumption, thus gradually contributing to AMR emergence and occurrence of antibiotic-related adverse events.

To the best of our knowledge, the vast majority of studies assessing ADE value were conducted in ICU settings [14,15,16,17,18,19,20,21,22], and little is, so far, known about ADE safety and efficacy in patients hospitalized in common wards. The aim of the present study was to evaluate the feasibility, safety, and efficacy of ADE in patients with microbiologically documented infection admitted to Greek medical wards with recognized prevalence of MDR pathogens.

## 2. Results

A total of 142 patients (male: *n* = 75, 52.8%) fulfilled the inclusion criteria and were analyzed. Mean age was 73.7 years (SD: 15.5) and median Charlson’s Comorbidity Index (CCI) was 5 (IQR: 5, 7). One in three patients (*n* = 46, 32.4%) reported an antimicrobials receipt in the last trimester for a median of two courses (IQR: 1, 6). On admission, the majority of the study population presented with sepsis (*n* = 80, 56.3%), and 18 patients were in septic shock (12.7%); their median Sequential Organ Failure Assessment (SOFA) and quick Sequential Organ Failure Assessment (qSOFA) scores were 3 (IQR: 1, 4) and 1 (IQR: 0, 1), respectively. The urinary tract was the most common site of infection (*n* = 95, 69.9%), followed by the lung (*n* = 19, 13.4%) and intra-abdominal sources (*n* = 8, 5.6%). About half of the infections (*n* = 66, 46.5%) were bacteraemic, and the incidence of primary bacteremia was 6.3% (*n* = 9). *E. coli* (*n* = 72, 50.7%), *K. pneumoniae* (*n* = 19, 13.4%), and *P. aeurginosa* (*n* = 11, 7.8%) were the predominant pathogens identified, and the most frequent sources of isolation were the urinary tract (*n* = 86, 60.6%) and the bloodstream (*n* = 71, 52.2%). Detailed patients’ characteristics classified using ADE status are depicted in Table 1.

The median time from hospital admission to the antimicrobial susceptibility testing report was 3 days (IQR: 3, 4), and ADE was applied in 76 patients (53.5%) at a median time of 4 days (IQR: 3, 5). The rest of the cohort was managed with either the initial (*n* = 29, 20.4%) or escalated (*n* = 37, 26.1%) antimicrobial therapy. The reported reasons for which ADE was not performed were as follows: (i) non-permissive antimicrobial susceptibility testing (*n* = 11, 16.7%), (ii) patient’s non-clinical improvement (*n* = 9, 13.6%), (iii) occurrence of a new infection (*n* = 4, 6.1%), (iv) type of isolated pathogen (*n* = 2, 3%), and (v) physician’s decision (*n* = 40, 60.6%).

In the multivariable logistic regression analysis (Table 2), upon adjustment for several confounders (e.g., sex, residence type, symptoms, SOFA score on admission, and source of infection), UTIs (OR: 10.04, 95% CI: 2.91, 34.57; *p* < 0.001), skin and soft tissue infections (SSTIs) (OR: 16.28, 95% CI: 1.68, 158.08; *p* = 0.016), and bloodstream infections (BSIs) (OR: 2.52, 95% CI: 1, 6.36; *p* = 0.05) were associated with an increased likelihood of ADE. Moreover, ADE was more likely to be applied in female patients (OR: 2.65, 95% CI: 1.09, 6.43; *p* = 0.031) and those that reported symptoms other than fever and disorder of consciousness on admission (OR: 3.44, 95% CI: 1.37, 8.67; *p* = 0.009).

**Figure 1:**
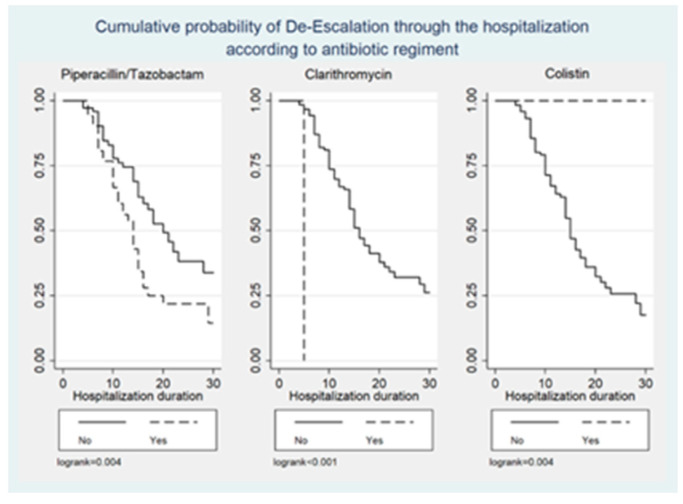
Cumulative probability of ADE by initial antimicrobial regimens administered.

**Figure 2:**
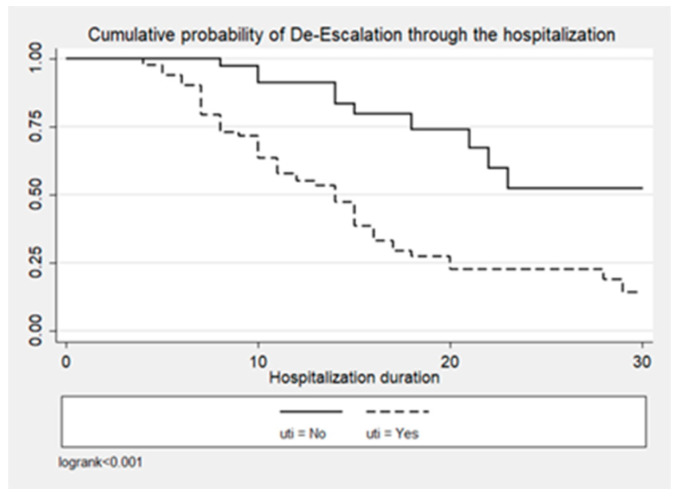
Cumulative probability of ADE by UTI as source of infection.

**Figure 3:**
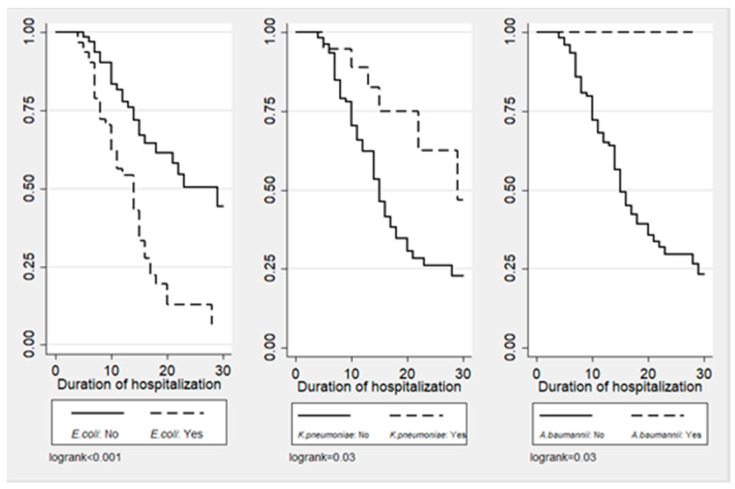
Cumulative probability of ADE by type of isolated pathogen.

Figure 1, Figure 2 and Figure 3 illustrate the cumulative probability of ADE by initial antimicrobial regimens administered, UTI as a source of infection, and isolated pathogens, respectively. UTI as a source had a higher cumulative probability of ADE compared to all other sources grouped together. Regimens containing clarithromycin had the highest, whereas regimens containing colistin had the least cumulative probability of ADE. Similarly, recovery of *E. coli* was more likely to be associated with ADE compared to *K. pneumoniae* and *A. baumannii*.

**Figure 4:**
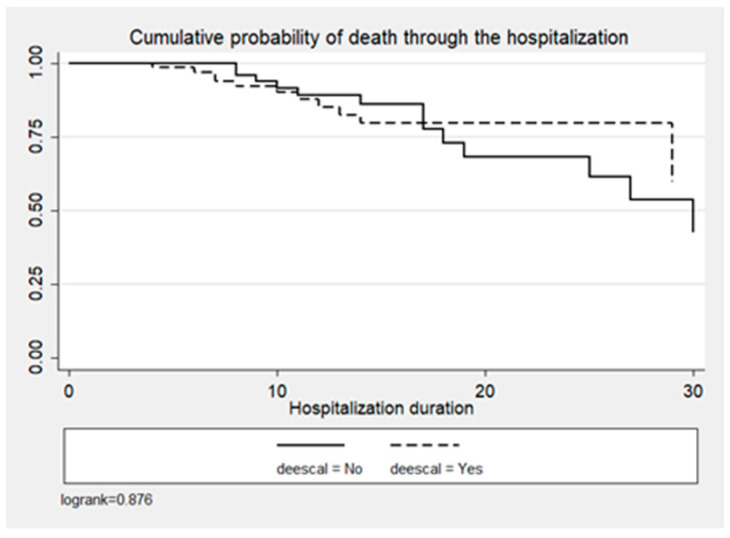
Cumulative probability of death by time since admission and DE decision.

In the entire cohort, crude mortality was 23.9% (n = 34), 18.4% (n = 14) for the ADE group vs. 30.3% (n = 20) for the non-ADE group (*p* < 0.1) (Table 1). ADE was not significantly associated with the probability of death (*p* = 0.432), as shown in Figure 4 and Table 3.

Mean length of hospital stay (LOS) was 12.5 (IQR: 8, 16) days and was significantly lower in the ADE group (11, IQR: 7, 15 vs. 14 IQR: 8, 19; *p* = 0.047). Antimicrobial therapy lasted for a median of 12.5 days (IQR: 10, 15) [ADE group: 12 days (IQR: 10, 14) vs. non-ADE group: 13 days (IQR: 9, 15); *p* = 0.9]. At the 30-day follow up, patients were antibiotic-free for a median of 4 days (IQR: 0, 14), and no difference was reported between the ADE and non-ADE groups (Table 1). Stress doses of hydrocortisone were administered in ten patients (median duration: 3.5 days, IQR: 3, 3.5) (Table 1). Vasopressor therapy was started on the third day of hospitalization in five (3.52%), on the ADE day in four (2.82%), on the third day upon ADE initiation in two (1.41%), and on day 28 in three (2.11%) patients, and the median duration of the therapy was 3.5 days (IQR: 2, 4.5) (Table 1). The need for both hydrocortisone and vasopressors administration was shown to be independent of ADE. Superinfection was observed in nine patients, of whom two were diagnosed with CDI, and XDR bacteria colonization was reported in two others. Acute kidney injury (AKI) emerged in 6.3% *(n* = 9) and multi-organ failure in 4.2% (*n* = 6), regardless of ADE status.

## 3. Discussion

The present study evaluated the feasibility, safety, and efficacy of ADE in patients with microbiologically documented infection admitted to medical wards with recognized prevalence of MDR pathogens. ADE was applied in the majority of the study population at a median of 4 days, which corresponds to a time frame that allows for the reporting of most diagnostic results and the assessment of a patient’s clinical response to empiric therapy [11,23]. The diagnosis of UTI, SSTI, and BSI was found to be predictive of ADE, thus suggesting that an ADE approach is appropriate and can be generally performed, as shown in previously published research [24,25,26,27,28,29,30]. Within the factors strongly associated with the probability of ADE, clarithromycin administration in patients with bacteremia or pneumonia conferred the highest ADE rate. This finding should not be surprising, since the addition of clarithromycin to the treatment regimen is well acknowledged to lead to better survival rates in patients with severe community-acquired pneumonia (CAP), decrease sepsis recurrence, and shorten time to recovery [31,32,33,34]. Most of the participating hospitals are members of a large sepsis study group, with a series of publications verifying the beneficial-immunomodulatory role of clarithromycin as adjunctive treatment in patients with CAP and nosocomial pneumonia, as well as sepsis caused by Gram-negative pathogens [31,32,33,34,35,36,37]. Furthermore, mortality rate, as well as median LOS, was lower in the ADE group, and the probability of death was proved to be independent of ADE, demonstrating that safety was not compromised. Lastly, the profile of the patient most eligible for ADE was delineated, mainly referring to a patient with clinical stability, sepsis from a UTI source and mostly caused by *E. coli*, or sepsis with symptoms and signs excluding an unknown origin. Profiling the eligible patient for ADE is an important tool for the local AMS team.

Our findings are in-line with previously published data reporting the favorable effect of ADE on mortality. A total of two randomized control trials (RCT) and twelve cohort studies evaluating ADE in critical care settings were included in the meta-analysis performed by Tabah et al., and the pooled estimated mortality was shown to favor ADE (relative risk [RR] 0.68, 95% CI: 0.52–0.88) [8]. The ADE effect on 30-day all-cause mortality in patients with BSI or pneumonia was addressed by Paul et al., and 19 studies (RCT: *n* = 3; observational studies: *n* = 16) conducted in non-ICU and ICU settings were analyzed. In approximately 4000 patients, mortality did not differ between the ADE group and the group in which the empiric antimicrobial therapy was maintained (OR: 0.83, 95% CI: 0.59–1.16) [27]. Ohji et al. sought to evaluate ADE effectiveness and safety for various types of infection by measuring the all-cause mortality. In the subset of patients experiencing community- or ICU-acquired pneumonia, as in the previous study, the 30-day mortality rate was significantly lower in the ADE group compared to the control group (OR: 0.50, 95% CI: 0.29–0.87; *p* = 0.01 and OR: 0.34, 95% CI: 0.17–0.68; *p* = 0.002, respectively), whereas it did not differ among those diagnosed with BSI, UTI, and ventilator-associated pneumonia or among those presenting with septic shock. Of note, the authors commented on the absence of high-quality evidence [29]. A trend towards lower mortality rate (RR: 0.74, 95% CI: 0.54–1.03; *p* = 0.005) was reported in patients with severe sepsis or septic shock in whom ADE was applied in the meta-analysis designed by Guo et al. [38]. The DIANA study, a prospective cohort study conducted in 152 ICUs across 28 countries, attempted to assess the feasibility of ADE in 1495 critically ill patients, as well as the ADE impact on clinical cure at day 7 following the empirical therapy initiation. No difference in the 28-day mortality rate was found between the ADE and no-ADE group, but there was a 30% increased likelihood of clinical cure at day 7 in de-escalated patients (RR: 1.32, 95% CI: 1.14–1.64) [14]. A recent publication of our group in the ICU setting showed that albeit ADE was feasible in a small percentage of septic ICU patients due to the antibiogram of the infecting pathogen, when applied, it was associated with lower all-cause 28-day mortality (13.3% vs. 36.7%, OR 0.27, 95% CI 0.11–0.66, *p* = 0.006) with a subsequent collateral decrease in the SOFA score. ICU and hospital mortality were also lower. Furthermore, a Cox multivariable regression analysis revealed that ADE was a significant factor for 28-day survival (HR 0.31, 95% CI 0.14–0.70, *p* = 0.005) [15]. Lastly, in several other studies conducted in the emergency room department, ICU setting, or medical ward, ADE did not confer increased mortality rates [19,25,28,39,40,41].

Although ADE does not seem to have a measurable impact on LOS in the literature, in our study, it resulted in significantly shorter LOS. In the meta-analysis performed by Ambaras Khan et al. that involved more than 2000 patients diagnosed with pneumonia and admitted to an ICU, despite the low quality of evidence, a statistically significant LOS reduction was observed in the ADE group (mean reduction: 5.96 days; 95% CI −8.39, −3.52) [16]. In a retrospective analysis of 240 patients who received piperacillin/tazobactam and vancomycin with the most commonly documented indications being sepsis and pneumonia, median LOS was 4 days shorter in ADE patients (6 vs. 10 days, *p* = 0.0003) [28]. Similarly, in a prospective analysis of carbapenem prescriptions and ADE performance, ADE reduced the median hospital stay by five days (*p* = 0.030) [16]. On the contrary, in several other studies and meta-analyses, including a non-blinded RCT, no changes in LOS were observed based on ADE status [14,20,27,40].

As a cornerstone of AMS strategies, ADE appears to be a safe policy for the prudent use of antimicrobials in patients with microbiologically documented infection based on the aforementioned. Nevertheless, robust data are lacking regarding the safety of ADE in patients with suspected infection and negative cultures. In the study of Moehring, R. et al., an antibiotic opt-out protocol was implemented as a randomized controlled trial, to decrease unnecessary antimicrobial use in patients with suspected sepsis. The study was conducted in ten Unites States (US) hospitals from September 2018 through May 2020 on non-ICU patients on broad spectrum antibiotics with negative blood cultures. The authors used an exhaustive 23-item safety check-list to ensure the safety of patients when proceeding with the opt-out randomization. Despite that, only antibiotics were stopped in only 59 out of 383 patients in the intervention arm, whereas in 299 patients, the treating physicians responded with the opt-out strategy. The treating physician’s belief that stopping antibiotics was unsafe (31%) was the second most common reason after treatment of local infection (76%) for antibiotic continuation. The length of therapy was similar in both the intervention and the control group, albeit the exposure of patients in the rank-3 agent group (extended spectrum) was statistically lower in the intervention group [42]. These data show that even under optimal conditions, antibiotic de-escalation has to overcome hurdles based on the physicians’ behaviors and established beliefs. In our opinion, in real practice, creating a similar safety check-list based on clinical and laboratory parameters which are locally available is an important tool in the hands of the AMS team. De-escalating antibiotics in patients with negative blood cultures, particularly in settings where molecular diagnostics are already available, may be feasible and safe as long as the clinical parameters are stable or ameliorating and regularly used biomarkers are in the same direction. However, the building of trust between the members of the team and the treating physicians remains the milestone for the success of these programs.

Regarding the rest of the exploratory end-points, the median duration of antimicrobial therapy was almost the same between the two groups. In the literature, antibiotic treatment duration varied and was reportedly decreased [17,19], similar [18,20], or longer in patients undergoing ADE [14]. Antimicrobial therapy duration in cases of ADE implementation is of major importance and should be carefully assessed [43]. Longer courses of antimicrobial therapy may result in increased incidence of MDR pathogens and adverse events, such as superinfections and CDI. On the other hand, shorter antimicrobial courses can be safely utilized in hospitalized patients with common infections (e.g., UTIs, intra-abdominal, or respiratory tract infections) to achieve clinical and microbiological resolution without affecting mortality or recurrence rates [44].

The rate of superinfections remained low in our cohort (*n* = 9, 6.3%) and was slightly higher in patients undergoing ADE (*n* = 5 (6.6%) vs. *n* = 4 (6.1%); *p* = 0.3). An increased incidence of superinfection in the ADE group (*n* = 16 (27%) vs. *n* = 6 (11%); *p* = 0.003) was also recorded by Leone et al. [40], whereas no difference in superinfection rates between ADE and empirical treatment continuation groups was found in other observational studies [21,22]. The increased occurrence of superinfections, observed both in our study and the RCT conducted by Leone et al., should be interpreted with caution, and the small number of patients experiencing a new infection should be taken into account [40].

There is a paucity of data regarding the effect of ADE on rates of *C. difficile* infection (CDI). It is only evaluated in a retrospective cohort study of 808 patients diagnosed with *Enterobacteriales* BSI and managed with antipseudomonal β-lactam (APBL) therapy for more or less than 48 h. The empirical use of APBL therapy > 48 h was found to be an independent risk factor for CDI [HR: 3.56, 95% CI: 1.48–9.92; *p* = 0.004], thus suggesting that early de-escalation of APBL using clinical risk assessment tools or rapid diagnostic testing may reduce the incidence of CDI [45].

Lastly, XDR pathogen colonization was rare in both patient groups, although it should be noted that patients were followed until hospital discharge, a time frame that may not be sufficient to assess the emergence, as well as the outcome, of MDR/XDR pathogens. To date, there is a lack of clinical data regarding the impact of ADE and AMR emergence. The few studies exploring AMR as an outcome reported no or a very limited effect of ADE on the individual or local prevalence of MDR/XDR bacteria [46]. In the DIANA study, a trend towards reduced MDR acquisition in the ADE group was noted (7.5% vs. 11.9; *p* = 0.06) [14]. Similarly, the acquisition rate of extended spectrum beta-lactamase (ESBL)-producing Enterobacterales in 182 patients with ventilator-associated pneumonia was lower in the ADE-group (1.4% vs. 8.2%; *p* = 0.07) [20]. In a prospective study on the evaluation of ASP-guided carbapenem ADE, a significantly lower incidence of carbapenem-resistant *A. baumannii* was revealed in the ADE-group (2% vs. 7.3%; *p* = 0.042) [47]. De Bus et al. sought to evaluate determinants of ADE and assessed whether the latter is associated with AMR emergence; in the analysis of 478 anti-pseudomonal beta-lactam prescriptions in tertiary ICUs, the risk of resistance emergence at day 14 was higher in the ADE group (30.6% vs. 23.5%; *p* = 0.22), but this may be partially attributed to the longer duration of treatment, which probably acted as a confounding factor [19]. In the analysis of Gonzalez et al., an increase in MDR bacteria colonization was detected in the ADE group (15.3% vs. 10.7; *p* = 0.1) of ICU patients treated for sepsis [48].

The findings of our study should be interpreted in light of certain limitations, such as the small number of patients analyzed, its observational character that automatically induces inclusion bias due to lack of adjustment for clinical stability, and the absence of a matched-comparison group to optimize the conclusions drawn. However, the fact that our data are in line with the previous publication regarding our group in the ICU setting supports the safety of DE in settings with a high prevalence of multidrug resistance despite compromised feasibility owing to the possibly MDR-infecting pathogens [15]. The study was conducted before the launch of new antibiotics (beta-lactams with beta lactamase inhibitors) in Greece and before the COVID-19 pandemic. As resistance issues seem to be on the rise during the pandemic years, the conclusions of our study can be used as a guide to local AMS teams in order to rekindle the battle against MDR pathogens not only in ICUs, but also in general wards [49,50].

## 4. Materials and Methods

### 4.1. Study Setting

This was a prospective observational cohort study conducted in the medical wards of 6 tertiary-care hospitals in Greece over a 2-year period (January 2017–December 2018). The participating centers [Attikon University General Hospital (Athens), General Hospital of Athens “G. Gennimatas” (Athens), Konstantopouleio General Hospital (Athens), Thriasio General Hospital of Elefsina (Attica), Laikon General Hospital (Athens), and “Papageorgiou” General Hospital of Thessaloniki (Thessaloniki)] are listed among the largest hospitals in Athens (Capital) and Thessaloniki (second most populated) metropolitan areas. The decision of de-escalation was at the discretion of the treating physicians. Local Antimicrobial Stewardship teams overlooked the antibiotic decisions, as per standard procedures.

### 4.2. Study Population and Data Collection

Adult patients aged ≥ 18 years with microbiologically documented infection, either at the time of hospital admission or acquired during their hospital stay, who were administered empirical antimicrobial therapy were analyzed. Patients in whom infection was suspected, but was not microbiologically confirmed, or in whom there was a lack of data regarding variables of interest, were excluded.

Data were prospectively extracted from routine care patient charts, including the following: demographic characteristics, CCI, residence type, receipt of antimicrobials in the past trimester, admitting diagnosis, illness severity assessed by SOFA and qSOFA scores, presence of sepsis or septic shock on admission and at preselected time points during hospitalization, vasopressors and/or hydrocortisone administration on admission and during hospitalization, development of AKI during or after the septic episode, and LOS. Other variables recorded were as follows: site of the microbiologically confirmed infection, identified pathogen, specimens of pathogen isolation, antimicrobial susceptibility testing report, empirical antimicrobial therapy administered, time interval from empirical antibiotic therapy initiation to applied ADE, duration of antimicrobial therapy, new episodes of infection (including CDI), and colonization with XDR pathogens.

Each participating institute was responsible for the data entry of its patients. Three of the authors (V.R., G.P., and H.G.) were responsible for the evaluation of the quality of entered data, data interpretation in cases of uncertainty, and communication with physicians of participating centers for additional information.

### 4.3. Definitions

Sepsis was defined as a life-threatening organ dysfunction caused by a dysregulated host response to an infection. It was presumed in patients with suspected or confirmed infection and SOFA score ≥ 2 or a SOFA score change ≥ 2 [51]. Septic shock was defined as a subset of sepsis in which profound circulatory, cellular, and metabolic abnormalities are associated with a greater risk of mortality than with sepsis alone. It was presumed in patients presenting with (i) persisting hypotension and requiring vasopressors to maintain mean arterial pressure (MAP) > 65 mmHg and/or (ii) blood lactate > 2 mmol/L despite adequate fluid resuscitation [51].

The site of infection was assessed according to criteria proposed by the US Center for Disease Control and Prevention [52].

Antibiotic-resistant pathogens were classified as MDR if they were non-susceptible to at least one agent in three or more antimicrobial categories, as extensively drug-resistant (XDR) if they were non-susceptible to at least one agent in all but two or fewer antimicrobial categories, and as pandrug-resistant (PDR) if they were non-susceptible to all agents in the available antimicrobial categories [53].

ADE was defined as the discontinuation of one or more components of combination empirical therapy and/or the change from a broad-spectrum to a narrower spectrum antimicrobial [7,8]. “Antimicrobial escalation” was defined as the addition of or switch to an antibiotic with a broader spectrum of activity. “No change” in antibiotic regimens administered was defined as the maintenance of the initial empirical antimicrobial therapy. For the purposes of the study, the patients were divided into two groups: the ADE group, in which DE was applied, and the non-ADE group, in which either the empirical antimicrobial therapy was maintained or escalation was performed.

Primary bacteremia was defined as the bacteremia for which no source of infection was documented. Bacteremia was defined as secondary when laboratory examination revealed infection by the same microorganism at a distant site in the same time or up to three days earlier.

AKI was defined by increased serum creatinine levels and reduced urinary output lasting a maximum of 7 days [54].

Superinfection was defined as an infection following a previous one, especially when caused by microorganisms that were resistant or have become resistant to the antibiotics used against the first infection [55].

### 4.4. Outcomes

We sought to assess the safety and efficacy of ADE in medical wards with recognized prevalence of MDR pathogens. Therefore, this study’s primary exploratory endpoints were: (i) the rate and feasibility of ADE in patients with microbiologically documented infection, (ii) the factors associated with ADE decision, and (iii) mortality in ADE vs. non-ADE groups. Secondary endpoints included duration of antimicrobial therapy, antibiotic-free days, need for vasopressors or/and hydrocortisone administration, incidence of new infections, including *C. difficile* and fungal infections, XDR pathogen colonization, development of AKI after the septic episode, and LOS.

### 4.5. Statistical Analysis

Baseline characteristics were summarized using descriptive statistics, including mean and standard deviation (SD) for normally distributed variables, medians and interquartile ranges (IQRs) for non-normally distributed variables, and absolute (N) and relative (%) frequencies for categorical ones. *T*-test and Mann–Whitney tests were used to compare continuous variables, while chi-square and Fisher’s exact tests were used for the categorical ones.

Survival analysis techniques, including multivariable Cox proportional hazards models, log-rank tests, and Kaplan–Meier curves, were conducted to evaluate the association of de-escalation to survival within 30 days upon hospital admission. Cumulative probability of recovery is presented as median (95% CI) time.

Factors possibly associated with DE were evaluated via multiple logistic regression models. Moreover, DE as decision was also evaluated as an outcome using the Kaplan–Meier estimator, and possible associations of other factors with DE decision were analyzed through Cox proportional hazard models.

STATA/MP13 (Stata Corp., College Station, TX, USA) was used for the analyses performed, and statistical significance was set at the 0.05 level.

### 4.6. Ethical Issues

This study was supported by the Hellenic Society of Chemotherapy. The protocol was approved by the institutional review boards of the participating hospitals and was conducted in accordance with the Helsinki Declaration of Human Rights. In compliance with the local regulations, the use of an informed consent form was waived because of the non-interventional character of the study and the use of anonymous clinical data in the analysis.

## 5. Conclusions

In the present study, patients with microbiologically documented infection admitted to Greek medical wards with recognized prevalence of MDR bacteria were prone to ADE, and when ADE was eventually applied, it was associated with favorable outcomes. Although no causality can be demonstrated, careful clinical selection of patients can result in a reduction in unnecessary antibiotic consumption without compromising safety. Our observations strengthen the importance of striving the process of de-escalation as part of ASP, even in areas with prevalent MDR. Exploitation of locally produced data can lead to the recognition of profiles of eligible patients and safely guide the efforts of the relevant antimicrobial stewardship teams.

Antibiotic overuse, irrespective of antibiotic class, is integrally linked to AMR, and the implementation of ASP and ADE is crucial. Well-designed targeted studies are needed to understand the benefits and risks of ADE in patients admitted to medical settings. An efficient ASP should incorporate a wide range of measures, including (i) optimization of empiric therapy with infection-site-specific guidelines, (ii) up-to-date local resistance data and assessments of the individual risks of resistant pathogens, (iii) review at the second or third empirical antimicrobial therapy with relevant microbiology results and clinical progress, and (iv) early cessation of antibiotics in unproven infection. Involvement of an infection specialist or pharmacist is beneficial, and rapid diagnostics may play a pivotal role [45].

## Figures and Tables

**Table 1:** Study population characteristics classified by de-escalation status.

Variables	ADE Group n = 76	Non-ADE Group n = 66	Overall n = 142	*p*-Value
*Baseline Characteristics*				
Mean age, years (SD)	74.5 (14.7)	72.8, (16.5)	73.7 (15.5)	0.664
Gender				0.024
Male *(%)*	33 (43.4)	42 (63.6)	75 (52.8)	
CCI (median, IQR)	5 (4, 7)	5 (3,7)	5 (3, 7)	0.542
Residence type, *n (%)*				0.068
Home	52 (68.4)	30 (45.5)	82 (57.7)	
Medical Ward	2 (2.6)	3 (4.5)	5 (3.5)	
ICU	0 (0)	1 (1.5)	1 (0.7)	
Healthcare facility	2 (2.6)	5(7.6)	7 (4.9)	
Long-term care facility	4 (5.3)	9 (13.6)	13 (9.2)	
Home with at least 1 hospitalization > 48 h since the last trimester	16 (21.1)	18 (27.3)	34 (24)	
Receipt of antimicrobials in the past trimester *(%)*	26 (34.2)	20 (30.3)	46 (32.4)	0.193
Times of antimicrobials receipt (median, IQR)	2.5 (2, 6)	2 (1, 6)	2 (1, 6)	0.378
*Symptoms on admission*, *n (%)*				
Fever	54 (71)	46 (69.7)	100 (70.4)	0.860
Disorder of Consciousness	19 (25)	11 (16.7)	30 (21.1)	0.681
Other symptoms	47 (61.8)	29 (43.9)	76 (53.5)	0.033
*Illness severity on admission*				
SOFA score (median, IQR)	3 (2, 4)	2 (1, 3.5)	3 (1, 4)	0.028
q-SOFA Score (median, IQR)	1 (0, 1)	1 (0, 1)	1 (0, 1)	0.603
Patients with sepsis, *n (%)*	44 (57.9)	36 (54.5)	80 (56.3)	0.622
Patients in septic shock, *n (%)*	11 (14.5)	7 (10.6)	18 (12.7)	0.452
*Type of Infection, n (%)*				
UTI	60 (78.9)	35 (53)	95 (66.9)	0.001
LRTI	3 (3.9)	6 (9.1)	9 (6.3)	0.196
CAP	4 (5.3)	5 (7.6)	9 (6.3)	0.580
HAP	0	1 (1.5)	1 (0.7)	0.283
Osteomyelitis	0	2 (3)	2 (1.4)	0.122
SSTI	3 (3.9)	3 (4.5)	6 (4.2)	0.840
Abdominal	5 (6.6)	3 (4.5)	8 (5.6)	0.606
BSI	39 (51.3)	27 (40.9)	66 (46.5)	0.139
Primary	3 (3.9)	7 (10.6)	10 (7)	0.233
Secondary	36 (47.4)	20 (30.3)	56 (39.4)	0.027
CLABSI	2 (2.6)	3 (4.5)	5 (3.5)	0.881
*Pathogen, n (%)*				
*Escherichia coli*	46 (60.5)	26 (39.4)	72 (50.7)	0.012
*Klebsiella pneumoniae*	8 (10.5)	11 (16.7)	19 (13.4)	0.284
*Pseudomonas aeruginosa*	4 (5.3)	7 (10.6)	11 (7.8)	0.235
*Proteus mirabilis*	4 (5.3)	4 (6)	8 (5.6)	0.837
*Acinetobacter baumannii*	0	5 (7.6)	5 (3.5)	0.015
Other	16 (21)	13 (19.7)	29 (20.4)	0.842
*Clinical specimens of pathogen isolation, n (%)*				
Blood	42 (58.3)	29 (45.3)	71 (52.2)	0.129
Urine	47 (61.8)	39 (59)	86 (60.6)	0.738
Sputum	1 (1.4)	3 (4.6)	4 (2.9)	0.257
Skin and soft-tissue	0	2 (3.1)	2 (1.4)	0.129
Other	5 (6.6)	3 (4.5)	8 (5.6)	0.600
*Empirical antimicrobial therapy*				0.064
Ampicillin-sulbactam or Ampicillin/sulbactam- based antimicrobial therapy	3 (3.9)	8 (12.1)	11 (7.7)	
Fluoroquinolones monotherapy or Fluoroquinolones-based antimicrobial therapy	6 (7.9)	12 (18.2)	18 (12.7)	
Piperacillin/tazobactam monotherapy or Piperacillin/tazobactam-based antimicrobial therapy	39 (51.3)	17 (25.7)	56 (39.4)	
Meropenem or Meropenem-based antimicrobial therapy	11 (14.5)	5 (7.6)	16 (11.3)	
Other	17 (22.4)	24 (36.4)	41 (28.9)	
Amikacin co-administration	29 (38.1)	15 (22.7)	44 (31)	
Vancomycin co-administration	15 (19.7)	8 (12.1)	23 (16.2)	
Metronidazole co-administration	4 (5.3)	1 (1.5)	5 (3.5)	
*Exploratory end-points*				
Mortality *(n, %)*	14 (18.4)	20 (30.3)	34 (23.9)	<0.1
LOS (median, IQR)	11 (7–15)	14 (8–19)	12.5 (8, 6)	0.047
Duration of antibiotic treatment (median, IQR)	12 (10, 14)	13 (9, 15)	12.5 (10, 15)	0.9
Antibiotic-free days (median, IQR)	4 (0, 14)	4(0, 14)	4 (0, 14)	0.8
Hydrocortisone administration (median, IQR)	0 (0, 0)	0(0, 0)	3.5 (3, 3.5)	0.7
Vasopressors administration (median, IQR)	0 (0, 0)	0(0, 0)	3.5 (2, 4.5)	0.3
AKI	3 (3.9)	6 (9.1)	9 (6.3)	0.6
Multi-organ failure	4 (5.3)	2 (3)	6 (4.2)	0.5
Superinfection	5 (6.6)	4 (6.1)	9 (6.3)	0.3
*Clostridium difficile* infection	1 (1.3)	1 (1.5)	2 (1.4)	0.9
XDR bacteria colonization	1 (1.3)	1 (1.5)	2 (1.4)	0.9

**ADE**: antimicrobial de-escalation; **AKI**: acute kidney injury; **BSI**: bloodstream infection; **CAP** = community-acquired pneumonia; **CCI**: Charlson’s comorbidity index; **CLABSI**: central line-associated bloodstream infection; **HAP** = hospital-acquired pneumonia; **ICU**: Intensive Care Unit; **IQR**: interquartile ranges; **LOS**: length of hospital stay; **LRTI**: lower respiratory tract infection; **SOFA**: sequential organ failure assessment; **SSTI**: skin and soft tissue infection; **qSOFA**: quick sequential organ failure assessment; **UTI**: urinary tract infection; **XDR**: extensively drug-resistant. **Note 1**: A subset of patients received empirical antimicrobial therapy for two concurrent documented infections; thus, the sum of both number of patients and percentages in the variable “type of infection” exceeded 100 and 100%, respectively. **Note 2**: Length of hospital stay, duration of antibiotic treatment, and median time of hydrocortisone and vasopressors administration are reported in days.

**Table 2:** Multivariable logistic regression analysis of factors associated with ADE.

	OR	95% CI	*p*-Value
Females vs. males	2.65	1.09–6.43	0.031
Symptoms on admission other than fever and disorder of consciousness	3.44	1.37–8.67	0.009
Previously been at home	2.43	0.10–5.92	0.051
SOFA on admission	1.22	1.00–1.51	0.064
UTI	10.04	2.91–34.57	<0.001
SSTI	16.28	1.68–158.08	0.016
Abdominal Infection	5.50	0.82–36.74	0.078
BSI	2.52	1.00–6.36	0.05

**BSI**: bloodstream infection; **CI**: confidence interval; **OR**: odds ratio; **SOFA**: sequential organ failure assessment; **SSTI**: skin and soft tissue infection; **UTI**: urinary tract infection.

**Table 3:** Multivariable Cox proportional hazards model for the probability of death.

	HR	95% CI	*p*-Value
			0.0002
Age	1.01	1.00–1.09	0.031
ADE performance	1.49	0.55–4.01	0.432
Moxifloxacin administration	36.50	2.39–556.6	0.010
Piperacillin/Tazobactam administration	4.36	1.47–12.99	0.008
Cefuroxime administration	26.49	5.75–122	<0.001

**CI**: confidence interval; **ADE**: antimicrobial de-esclation; **HR**: hazard ratio.

## Data Availability

Raw data is not available due to patient confidentiality, but can be provided upon request.
